# Valorization of Brewers’ Spent Grain for the Production of Lipids by Oleaginous Yeast

**DOI:** 10.3390/molecules23123052

**Published:** 2018-11-22

**Authors:** Alok Patel, Fabio Mikes, Saskja Bühler, Leonidas Matsakas

**Affiliations:** Biochemical Process Engineering, Division of Chemical Engineering, Department of Civil, Environmental and Natural Resources Engineering, Luleå University of Technology, 971-87 Luleå, Sweden; alok.kumar.patel@ltu.se (A.P.); fabio.mikes@gmail.com (F.M.); saskja.buhler@ltu.se (S.B.)

**Keywords:** Brewer’s spent grain, oleaginous yeast, *Rhodosporidium toruloides*, organosolv, biodiesel

## Abstract

Brewers’ spent grain (BSG) accounts for 85% of the total amount of by-products generated by the brewing industries. BSG is a lignocellulosic biomass that is rich in proteins, lipids, minerals, and vitamins. In the present study, BSG was subjected to pretreatment by two different methods (microwave assisted alkaline pretreatment and organosolv) and was evaluated for the liberation of glucose and xylose during enzymatic saccharification trials. The highest amount of glucose (46.45 ± 1.43 g/L) and xylose (25.15 ± 1.36 g/L) were observed after enzymatic saccharification of the organosolv pretreated BSG. The glucose and xylose yield for the microwave assisted alkaline pretreated BSG were 34.86 ± 1.27 g/L and 16.54 ± 2.1 g/L, respectively. The hydrolysates from the organosolv pretreated BSG were used as substrate for the cultivation of the oleaginous yeast *Rhodosporidium toruloides,* aiming to produce microbial lipids. The yeast synthesized as high as 18.44 ± 0.96 g/L of cell dry weight and 10.41 ± 0.34 g/L lipids (lipid content of 56.45 ± 0.76%) when cultivated on BSG hydrolysate with a C/N ratio of 500. The cell dry weight, total lipid concentration and lipid content were higher compared to the results obtained when grown on synthetic media containing glucose, xylose or mixture of glucose and xylose. To the best of our knowledge, this is the first report using hydrolysates of organosolv pretreated BSG for the growth and lipid production of oleaginous yeast in literature. The lipid profile of this oleaginous yeast showed similar fatty acid contents to vegetable oils, which can result in good biodiesel properties of the produced biodiesel.

## 1. Introduction

Nowadays, the most significant challenge for researchers, environmentalists, and stakeholders is to clean our environment as it is rapidly deteriorating as a consequence of increasing demand of land, water, food, and energy [1]. Increasing public concerns about global warming and green-house gas emissions attributed to the use of fossil fuels have attracted the attention to these issues which are considered as a grand challenge in the 21st century [2]. Apart from the environmental concerns raised by using fossil resources for energy production, import of fossil fuels is also negatively impacting domestic economies due to supply insecurity. For any developed and developing nation it is crucial to uphold economical and sustainable growth with the utilization of domestic and renewable sources of energy so that the import of oils can be reduced [3]. In recent years, fuels produced from biomass-based resources have received a growing interest as they are among the most promising replacements for non-renewable fossil fuel energy [4]. However, utilization of edible sources as raw materials for biofuels production can directly compete with human foods and has therefore been criticized [5]. Hence, it is essential to explore novel, alternative, sustainable, and renewable feedstocks for energy production to satisfy the current and future energy requirement of our society.

Among the various alternatives of biomass-derived fuels, biodiesel is the most suitable for compression-ignition (diesel) engines [6]. Earlier, vegetable oils without transesterification were tested in this type of engines, but due to their higher viscosity they were not compatible for such operation [7]. While transesterification solved the problem of viscosity, debates on “food vs. fuel” obstruct the utilization of vegetable oil feed stocks for biodiesel production [5]. The oils obtained from oleaginous microbes can substitute the vegetable oils for biodiesel production. The microbial oils comprise an intracellular product of different kind of oleaginous microorganisms such as bacteria, yeasts, algae, and fungi [8]. Among the different classes of microorganisms, yeasts are considered to be best suitable for lipid production, which can be used as feed stock for the production of biodiesel [9]. Oleaginous yeasts present various positive characteristics such as independency from weather conditions, high lipid productivity and the ability to utilize a wide range of inexpensive and renewable waste substrates derived from agricultural and industrial practices, which makes them an excellent choice for lipid production and the substitution of oily crops [10]. The majority of oleaginous yeasts demonstrate a preference to utilize glucose over other carbon sources that might be present and will not shift to the other sugars until glucose is fully utilized [11]. This type of microorganisms have a carbon catabolite repression (CCR) mechanism that suppresses the utilization of other sugars when glucose is present and is responsible for the utilization of sugars in a sequential manner, which in turn is expressed as a delay in the growth when the microorganism is grown on a mixture of sugars [12]. To permit a financially sustainable commercial application of oleaginous yeasts within the biofuel market, their cultivation must be supported by inexpensive raw materials such as non-edible lignocellulosic feedstocks. The hydrolysates obtained from various non-edible lignocellulosic feedstocks often contain a mixture of pentoses and hexoses [13]. It is therefore economically advantageous for any process to incorporate the use of components in the lignocellulosic hydrolysates in a simultaneous manner. Unfortunately, co-utilization of various sugars by such microorganisms is rarely observed. However, certain oleaginous yeasts have the ability to utilize a mixture of sugar sources simultaneously. Oleaginous yeasts belonging to the *Rhodosporidium* genera have been extensively studied for the simultaneous utilization of mixtures of sugars derived from lignocellulosic biomass [14]. For this purpose, various strains belonging to the *Rhodosporidium* genus have been grown on different types of non-edible lignocellulosic biomass such as rice straw, wheat straw, corn powder, cassava, Jerusalem artichoke, sugarcane, sweet sorghum, waste molasses, soy whey, and glycerol, a byproduct from biodiesel industries [14].

Brewers’ spent grain (BSG) is the main by-product of the beer brewing industries, accounting for 85% of the total by-products generated [15,16]. The brewing process involves several stages, namely malting, milling, mashing, brewing, cooling and fermentation [17]. After the enzymatic conversion (mashing) of barley starch, the insoluble materials of grains are generally filtered from the sweet wort containing the fermentable sugars, generating the BSG [16]. For each 100 L of brewed beer produced, almost 20 kg of wet BSG is generated as a by-product [18]. In Europe alone, the generation of BSG reaches approximately 3.4 million tons per year [19], which is expected to further increase as a result of the blooming micro-brewery market. BSG is a lignocellulosic biomass that consists mainly of fibers such as cellulose and hemicellulose and other compounds such as protein, lipids, starch, and lignin [18]. The chemical composition of BSG is dependent on the type of barley grains, its harvesting time, the addition of other adjuncts, the composition of the hops, and of course the brewing technology [20,21]. Due to high moisture content together with high content in polysaccharides and proteins, BSG is prone to microbial contamination making its storage stability limited to up to 7–10 days [18]. This also has an impact on the transportation of BSG, which becomes expensive due to high-water content, resulting in the discharge of BSG in landfills being a common practice which can lead to environmental problems [16]. These characteristics reduce the commercial value of BSG with the use as livestock feed to be the most common practice aside landfilling for the treatment of BSG [16]. To improve the commercial value of BSG and reduce the environmental issues of the uncontrolled emission in landfills, there is a strong research interest in both, establishing techniques that will improve its storage stability and evaluating new valorization schemes that will yield high-added value. To this extent, various techniques have been applied for its storage and preservation [15], whereas applications such as human nutrition, energy production, charcoal production, adsorbents, and incorporation in biotechnological applications have been studied [22]. As for the biotechnological application, BSG has been studied as a substrate for enzyme production, biogas, ethanol fermentation, cultivation of mushrooms, and production of lactic acid [15].

The objective of the current work is to study the potential of incorporating BSG in the production of renewable energy in the form of biodiesel. For this purpose, we studied the growth and the lipid accumulation of the oleaginous yeast *Rhodosporidium toruloides*, a yeast capable of co-utilizing xylose and glucose, when using BSG as medium. As BSG is a lignocellulosic rich substrate, it presents a natural recalcitrance towards enzymatic depolymerization of the insoluble carbohydrates (cellulose and hemicellulose). To improve the release of soluble sugars from BSG, we incorporated a pretreatment step with organosolv and microwave assisted alkaline pretreatment prior to enzymatic saccharification.

## 2. Results and Discussion

### 2.1. Characterization and Enzymatic Hydrolysis of BSG

BSG is mainly composed of cellulose, hemicellulose and lignin along with considerable amounts of proteins and lipids and its composition varies depending on the applied brewing techniques [19]. The chemical composition of BSG along with its high fiber content enables it to serve as a potential feedstock for several commercial processes, including applications in biotechnology, thermochemical and biochemical engineering such as renewable energy, enzyme production and bread making, as well as production of ethanol, butanol, xylitol, activated carbon, charcoal and oligo saccharides production [17]. A comparative study of BSG composition from various sources including the BSG used in the present work is presented in Table 1. The composition of BSG used in this study (% *w*/*w*, dry basis) was cellulose (38.88 ± 0.87), xylan (16.23 ± 1.17), protein (12.54 ± 0.65), and lignin (13.7 ± 0.98).

**Figure 1:**
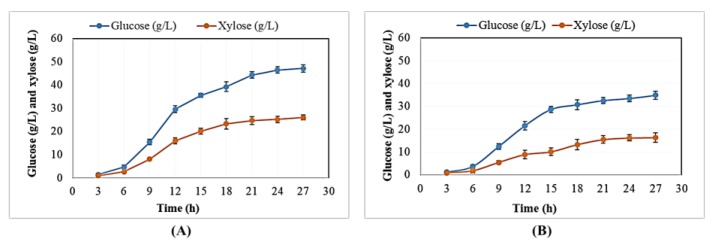
Glucose and xylose production during the enzymatic hydrolysis of organosolv pretreated Brewers’ spent grain (BSG) (**A**) and microwave assisted alkaline pretreated BSG (**B**).

To effectively incorporate BSG in a biotechnological application, a pretreatment step is often required, aiming to disrupt the natural recalcitrance of lignocellulose. As a result of the pretreatment, the polysaccharides are exposed and become easier saccharified by the action of cellulolytic enzymes. In this study, two different pretreatment strategies (microwave assisted alkaline pretreatment and organosolv) were evaluated on the ability to enhance the subsequent saccharification yields. The pretreated BSG was enzymatically hydrolyzed by using a mixture of the commercial enzyme solution Cellic CTec2 and Accellerase XY in a 5:1 ratio (*v/v*) and the rate of production of glucose and xylose is presented in Figure 1. As shown in Figure 1A, the amount of glucose and xylose production from the organosolv treated BSG increased up to 27 h of saccharification. The total amount of glucose reached 46.45 ± 1.43 g/L after 27 h of enzymatic saccharification. At the same time, the total amount of xylose was 25.15 ± 1.36 g/L. In the case of microwave assisted alkaline pretreated BSG, the values for glucose and xylose were lower compared to the organosolv pretreated BSG, reaching 34.86 ± 1.27 g/L and 16.54 ± 2.1 g/L, respectively, after 27 h of saccharification (Figure 1B). Due to the higher concentration of sugars obtained by the organosolv pretreated BSG, the hydrolysates from this pretreatment were used for the cultivation of the oleaginous yeast.

### 2.2. Optimization of the C/N (g/g) Ratio for Lipid Accumulation of R. Toruloides

Lignocellulosic biomass hydrolysates contain mainly two sugars, namely glucose and xylose, whereas other sugars such as mannose and arabinose might be present, depending on the source of biomass [26]. Glucose is the most preferred carbon source among such monomeric carbon sources by the majority of microorganisms [11]. The microorganisms do not shift to the consumption of other sugars until glucose is fully consumed. Co-utilization of glucose with other sugar sources is rarely observed, however, this strategy has potential to improve the economics of biofuel and value-added chemical production by microbial fermentation [13].

**Figure 2:**
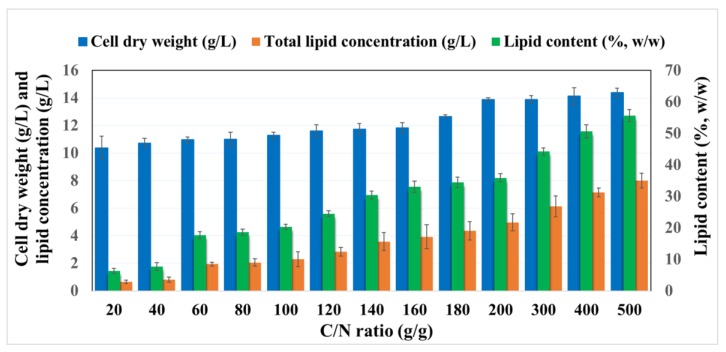
Effect of various C/N ratios (g/g) on the cell dry weight (g/L), lipid concentration (g/L) and lipid content (%, *w*/*w*) of *Rhodosporidium toruloides.*

**Figure 3:**
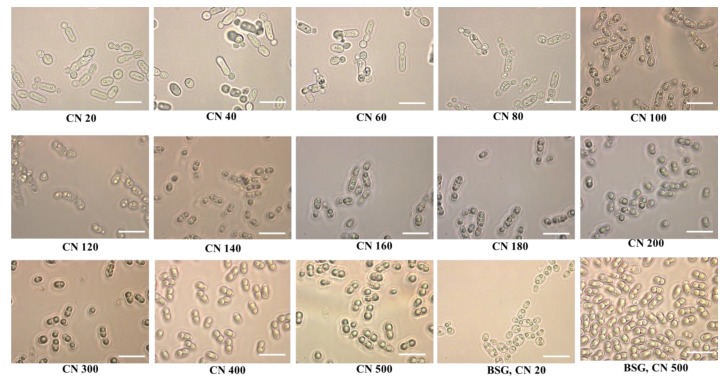
Representative images of cell morphology and lipid droplets synthesis in *R. toruloides* grown on a mixture of glucose (40 g/L) and xylose (20 g/L) at various C/N ratios and BSG hydrolysates (at C/N ratio of 20 and 500). The bar represents 10 μm.

The lipid accumulation by oleaginous microorganisms is not only dependent on the initial concentration of the provided carbon source but also differs depending on other media components and can be enhanced by keeping a key nutrient limited [27]. Key nutrients such as nitrogen, phosphorus and sulfur and their ratios with the carbon source (C/N, C/P, C/S) have been argued to have an important effect on the lipid accumulation [28,29,30]. For this purpose, prior to the cultivation of the yeast on the BSG hydrolysates, the C/N ratio was optimized with the aim to achieve high lipid accumulation in the yeast cells. The optimization experiment for the lipid accumulation in *R. toruloides* on synthetic media mimicking the composition of BSG hydrolysates was carried out with a mixture of 40 g/L of glucose and 20 g/L of xylose, as similar concentrations of glucose and xylose were observed in the organosolv pretreated BSG after enzymatic hydrolysis. The nitrogen concertation was adjusted by varying the amount of ammonium sulphate aiming to obtain different C/N (g/g) ratios ranging from 20 to 500. The cell dry weight (g/L), total lipid concentration (g/L) and lipid content (%, *w*/*w*) of *R. toruloides* grown on different C/N ratios are presented in Figure 2. An increase in cell dry weight from 10.38 ± 0.83 g/L to 14.39 ± 0.32 g/L along with a significant increase in lipid accumulation from 0.66 ± 0.12 g/L to 8.0 ± 0.54 g/L was observed when the C/N ratio was increased from 20 to 500 (Figure 2). Initially, the cultivation was studied in a range from C/N 20 to C/N 180 (g/g), however, the increasing lipid accumulation in the cellular compartment prompted us to check even higher C/N ratios ranging from 180 to 500. The cell dry weight presented only a minor increase from 13.88 ± 0.13 g/L to 14.39 ± 0.32 g/L when the C/N ratio increased from 200 to 500, however, a steep increase in lipid concentration from 4.97 ± 0.62 g/L to 8.00 ± 0.54 g/L was observed (Figure 2). The increase in cell dry weight was observed mainly due to the increased amount of lipids accumulated in the cellular compartment of the oleaginous yeast as presented in Figure 3. Large lipid droplets in the cellular compartment of the oleaginous yeast were observed with increasing C/N ratios (Figure 3). Lipid-free dry biomass decreased with increasing C/N ratio, which is also reported by other researchers [28,31].This is a result of a rapid decrease in intracellular AMP concentration as a consequence of nitrogen exhaustion from the medium, which affects the Krebs cycle function. The carbon flow is directed towards the synthesis of intra-mitochondrial citric acid due to the deactivation of isocitrate dehydrogenase as a result of low intracellular AMP [28]. High amounts of citric acid in the mitochondria are exchanged with malic acid, which is present in the cytoplasm, via the citrate/malate translocase present in the mitochondrial membrane. Citric acid is then cleaved into acetyl-CoA and oxaloacetate by the action of the ATP-citrate lyase. Finally, acetyl-Co-A and malonyl-Co-A react together to form fatty acid chains between C_14_ and C_16_ [32]. This mechanism is the reason why the excess of glucose in low nitrogen conditions is converted into fatty acids in oleaginous yeast [32,33,34]. However, in the case of conventional or non-oleaginous yeasts like *Saccharomyces cerevisiae* the excess carbon source under nitrogen-limited condition is converted into mannans and glucans [35].

### 2.3. Time Course Experiments of R. toruloides Grown in BSG Hydrolysates

**Figure 4:**
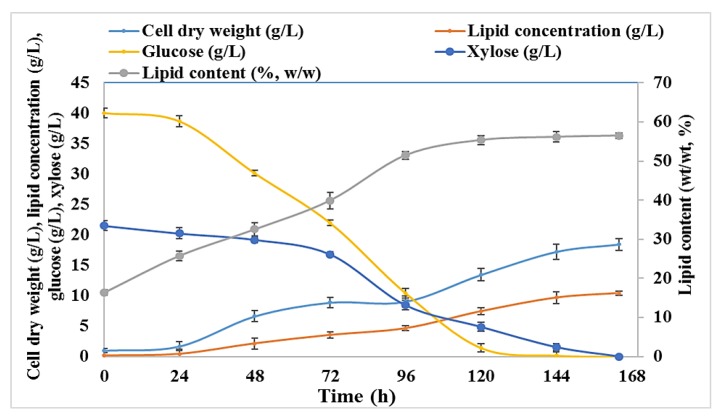
Time course experiments for cell dry weight (g/L), total lipid concentration (g/L), lipid content (% *w*/*w*) and residual glucose and xylose in *R. toruloides* grown for 168 h on BSG hydrolysate with C/N 500.

After the optimization of the C/N ratio to yield high lipid accumulation, the use of BSG hydrolysates was tested at an initial sugar concentration of 40 g/L glucose and 21.5 g/L xylose. The time course experiments of the cell dry weight (g/L), total lipid concentration (g/L), lipid content (%, *w*/*w*), residual glucose and xylose in medium are presented in Figure 4. The highest cell dry weight (18.44 ± 0.96 g/L) and lipid concentration (10.41 ± 0.15 g/L) were observed when cells were grown in BSG hydrolysate for 168 h. Almost all glucose (96.42%) had been consumed after120 h of cultivation, while only 77.34% of xylose had been utilized in the same period. The maximum utilization of xylose was observed when glucose levels were below 25 g/L. The lipid synthesis was minimal at the initial phase of cultivation as the increment of lipid accumulation from 16.32 ± 0.67% *w*/*w* to 39.90 ± 2.1% *w*/*w* was observed from 24 h to 72 h, however, a rapid increase in lipid accumulation was observed after 96 h of cultivation when xylose started to be consumed together with glucose. The enhancement in biomass compared to the synthetic media can be attributed to the presence of other nutrients in BSG hydrolysates (data not reported here).

Co-utilization of C6- and C5-sugar sources by oleaginous yeasts is a rare event and the consumption of various carbon sources is strain dependent as the same oleaginous yeast *R. toruloides* CCT 0783 has the ability to co-utilize glucose and fructose almost at the same consumption rate [36]. On the other hand, other strains of *R. toruloides* are reported to utilize sugars sequentially. For example *R. toruloides* ATCC 24196 consumes glucose at a faster rate than xylose, xylulose and xylitol [37]. Recently, *R. toruloides* DSM4444 was explored for the utilization of lignocellulosic-based sugars for lipid production [38]. This strain also showed sequential sugar utilization and consumption of xylose was observed when glucose levels were below 20 g/L [38]. Not only *Rhodosporodium* sp. but also other oleaginous yeast strains have the capability to consume glucose and xylose simultaneously, such as *Lipomyces starkeyi* AS 2.1560 cultivated on glucose and xylose (2:1, *w*/*w*) for lipid production [39]. Similarly, *Trichosporon fermentans* was grown on sulphuric acid treated rice straw hydrolysatecontaining glucose, xylose, mannose, galactose, and cellobiose and synthesized 10.4 g/L lipid in its cellular compartment [40]. *R. toruloides* Y2 was studied for lipid production by using waste nutrients from bioethanol waste water and synthesized 34.9% lipid content on the basis of cell dry weight while the lipid content was increased up to 53.8% on addition of glucose (1.2 g/L) [41].

### 2.4. Estimation of Extracted Fatty Acids and Transesterified Products by Thin Layer Chromatography

**Figure 5:**
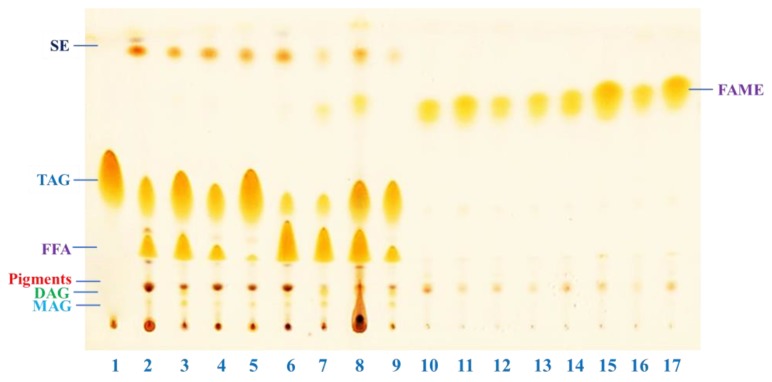
Separation of total lipid extracted from *R. toruloides* grown on 40 g/L glucose with C/N 20 (lane 2) and 500 (lane 3), 20 g/L xylose with C/N 20 (lane 4) and 500 (lane 5), mixture of glucose (40 g/L) and xylose (20 g/L) with C/N 20 (lane 6) and C/N 500 (lane 7), and BSG hydrolysate with C/N 20 (lane 8) and C/N 500 (lane 9). Glyceryl trioleate was used as standard for TAG (triacylglycerides) (lane 1). Corresponding fatty acid methyl esters after transesterification are spotted in lane 10 to lane 17.

A TLC (thin layer chromatography) chromatogram of the lipids extracted from oleaginous yeast grown on synthetic media and BSG hydrolysates at various C/N ratios is shown in Figure 5 (lane 2 to lane 9). The corresponding FAMEs (fatty acid methyl esters) after transesterification are presented in lanes 9 to 17 (Figure 5). High amounts of TAGs (triacylglycerides) were observed when yeast was grown in glucose or xylose under nitrogen limited condition (C/N 500) (lane 3 and 5, Figure 5). A dramatic decrement of TAGs with an increment of FFAs (free fatty acids) was observed when glucose and xylose were provided in a mixture (lane 6 and 7, Figure 5). On the other hand, high amounts of TAGs were observed in BSG hydrolysate under nitrogen-limited condition (C/N 500) as presented in lane 9 (Figure 5), while FFA content was increased at low C/N ratio (lane 8, Figure 5).

Oil feedstocks with a high amount of FFAs are not suitable for conversion into biodiesel using an alkaline catalyst [42]. Such oils require an esterification first with an acid catalyst to reduce the content of FFA in the feedstock prior to transesterification with a basic catalyst to complete the transesterification reaction [43]. Feedstocks with a high amount of TAGs are always a better choice for biodiesel production through transesterification, as in a complete reaction every mole of TAG produces three moles of biodiesel and one mole of glycerol [43]. Almost all TAGs and FFAs were converted into FAMEs by the transesterification reactions as shown in lane 10 to lane 17.

### 2.5. Fatty Acid Analysis of the Lipids Obtained from R. toruloides

The effect of different substrates, namely glucose, xylose and a mixture of glucose and xylose, and BSG hydrolysate along with different C/N ratios on the fatty acid profile of *R. toruloides* is represented in Table 2. It has already been reported that oleaginous yeasts grown in glucose-based medium synthesized mainly myristic acid (C_14:0_), palmitic acid (C_16:0_), stearic acid (C_18:0_), oleic acid (C_18:1_), along with linoleic acid (C_18:2_) and traces of linolenic acid (C_18:3_) [44]. However, it is of interest to examine how the fatty acid profile alters when the yeast grows on pure mixtures of glucose and xylose or on BSG hydrolysate. When *R. toruloides* was grown on 40 g/L glucose at C/N 20, the fatty acid content was C_16:0_ (13.33%), C_18:0_ (6.69%), C_18:1n9t_ (46.25%), C_18:2n6c_ (17.97%), C_18:3n3_ (1.43%), and C_22:2_ (1.12%) while in nitrogen-limited condition (C/N 500), the profile changed significantly to C_14:0_ (0.98%), C_16:0_ (21.52%), C_18:0_ (8.10%), C_18:1n9t_ (51.14%), C_18:2n6c_ (11.60%), C_18:3n3_(1.60%) and C_22:2_ (1.24%) (Table 2). The oleaginous yeast grown on 20 g/L of xylose at C/N 20 synthesized 26.87% of SFAs (saturated fatty acids), 43.45% of MUFAs (monounsaturated fatty acids) and 27.19% of PUFAs (polyunsaturated fatty acids) while the corresponding values at C/N 500 were 30.46%, 42.37% and 15.3%, respectively. When a mixture of glucose 40 g/L and xylose 20 g/L at C/N 20 was provided to *R. toruloides,* the SFAs, MUFAs, and PUFAs were 26.87%, 43.45%, and 27.19%, respectively, while at C/N 500 the corresponding values were 31.4%, 52.3%, and 14.31%, respectively. A similar trend was also observed when this yeast was grown on BSG hydrolysate, as 20.07% of SFAs were observed with C/N 20, while an increased value of SFAs (33.10%) was observed at C/N 500 (Table 2). Decreasing content of MUFAs from 54.84% to 50.85% and PUFAs from 24.84% to 13.97% were observed when *R. toruloides* was shifted from C/N 20 to C/N 500.

The biodiesel properties were theoretically estimated by using fatty acid profiles of oleaginous yeast grown on various substrates as listed in Table 2 and the data was compared to international standards such as ASTM 6751-3 (USA) and EN 14214 (Europe) (Table 3). Biodiesel is defined as a mixture of alkyl esters of the corresponding fatty acid profile of the parent oil, mainly containing carbon chains of C_16_ to C_18_. However, significant amounts of chain lengths other than C_16_-C_18_ have also been reported in some feedstocks such as tropical oils (e.g. coconut oil) enriched in shorter-chain acids such as lauric acid [45]. The LCSF (long chain saturation factor) is one of the most important criterion that is interconnected with the estimation of significant parameters of biodiesel such as oxidative stability, cetane number, viscosity and cold flow behavior [46]. The LCSF is affected by the chain length of the fatty acid, the number and position of the double bonds present in the fatty acids. A high value for LCSF gives a negative impact on the cold flow behavior of biodiesel. Cold flow behavior is measured by the CFPP (cold filter plugging point), which is a measurement of the lowest temperature where a fuel can easily pass through a standardized filtration unit in a specific time [47]. In cold environment, especially in Northern European countries, the low-temperature operability of biodiesel is a major problem due to crystallization of fuel in the pipeline of the engine [48,49]. Based on the data listed in Table 2, the values for LCSF were 7.978, 8.562, 8.456, 8.772, 5.517, 6.736, 3.405, and 8.060 for the biodiesel obtained from oleaginous yeast grown on 40 g/L glucose with C/N 20 and 500, 20 g/L xylose with C/N 20 and 500, mixture of glucose (40 g/L) and xylose (20 g/L) with C/N 20 and C/N 500, and BSG hydrolysate with C/N 20 and C/N 500, respectively, and the corresponding CFPP values were 8.6, 10.4, 10.1, 11.1, 0.9, 4.7, 0.6, and 8.8 °C. It is clear from the data that the biodiesel obtained from oleaginous yeast cultivated on BSG hydrolysate with C/N 20 has the lowest value of CFPP (0.6 °C) compared to the rest of the oils, which could be suitable for operation in cold environment.

Oxidative stability, which is dependent on the degree of unsaturation of a fatty acid, is another important parameter that determines the shelf-life of a fuel [50]. Biodiesel listed in Table 3 fulfills the criteria established by both standards, ASTM 6751-3 (minimum 3 h) and EN 14214 (minimum 6 h). The highest degree of oxidative stability (12.1 h) was observed when the oleaginous yeast was grown on BSG hydrolysate with C/N 500 (Table 3). Cetane numbers (CN) of all the biodiesels listed in Table 3 also meet the criteria stipulated by ASTM 6751-3 (minimum 47 min) and EN 14214 (minimum 51 min). CN has two limits, a lower and an upper one. Values above the upper limit reduce the engine efficiency due to improper mixing of air when starting the engine, while values below the low limit cause problems in low-temperature operation and result in high emissions of hydrocarbons [51]. The highest value of CN (61.8) was obtained with the biodiesel from the oleaginous yeast grown in 20 g/L xylose with C/N 20 while the lowest value (52.1) was obtained with BSG hydrolysate with C/N 20 (Table 3). It is clear from the above discussion, that high MUFA content is advantageous as it enhances the cold flow plugging properties (CFPP) while high SFA content gives a high value of oxidative stability. Therefore, it is important to control all biodiesel properties with the optimum ratio of SFA to MUFA in FAME [52]. Finally, the biodiesel produced from the lipids derived from the yeast growing on BSG hydrolysate presents a higher high heating value (38.7 MJ/Kg) compared to other biodiesels. The high heating value of some vegetable oils such as cotton seeds oil, coconut oil, and palm kernel oil were reported as 38.93, 37.54, and 37.68, respectively [53].

## 3. Materials and Methods

### 3.1. Collection of BSG and Characterization

All chemicals used in the present study were of analytical grade and were purchased from Sigma Aldrich (St. Louis, MO, USA). BSG was provided by a local microbrewery. The composition of BSG in cellulose, hemicellulose, and lignin was determined according to a two stage acid hydrolysis method described in the National Renewable Energy Laboratory (NREL) protocol [54]. The ash content of BSG was determined as the percentage of residue remaining after dry oxidation of biomass at 550 to 600 °C for 4 h [55]. The moisture content was determined gravimetrically after drying the samples in an oven at 105 °C until constant weight was reached. To reduce the moisture content of the wet BSG, it was dried in an oven at 60 °C for two days, followed by grinding it to a particle size of <3 mm by a commercial kitchen blender to improve the pretreatment efficiency. The grinded BSG was stored at room temperature until further use.

### 3.2. Pretreatment of BSG and Enzymatic Hydrolysis

In the present study, BSG was pretreated with two different methods to facilitate the increment of cellulose content in the pretreated solids and to maximize the release of sugars upon enzymatic hydrolysis. The first method involved a microwave assisted alkali pretreatment as described previously in the protocol of Ravindran et al. (2018) [56]. More specifically, a solution of 1% *w*/*v* dried BSG in 0.5% NaOH was prepared in a capped flask and subjected to microwave radiation in a domestic microwave at 400 W for 60 s. At the end of the treatment, the biomass was filtrated in a vacuum filtration unit and washed with distilled water until pH 6.0 was reached. Finally, the pretreated solids were air-dried on filter paper at room temperature. The dried solid residue was used for compositional analysis and enzymatic hydrolysis. In the second method, BSG was treated with organosolv pretreatment as previously described by our group [57]. In brief, 110 g of dry biomass were mixed with a 60% *v*/*v* ethanol in water solution with the addition of sulfuric acid (1 wt% on dry biomass) as acidic catalyst. Organosolv pretreatment took place at 175 °C for 60 min in metallic cylinders, which were placed in an air-heated multi-digester apparatus. At the end of the procedure, the pretreated solids were separated from the liquor by vacuum filtration, washed with ethanol and then air dried at room temperature.

Enzymatic saccharification of the pretreated BSG was carried out using the commercial enzyme solution Cellic CTec2 (Novozymes A/S, Bagsværd, Denmark) at a concentration equal to 20 FPU/g of solids. To enable better hydrolysis of the hemicellulose, Cellic CTec2 was supplemented with Accellerase XY (DuPont, Wilmington, Delaware, USA) at a ratio of 1:5 (*v*/*v*) of Cellic CTec2. The reaction was carried out in 500 mL Erlenmeyer flasks containing 100 g of 10% *w*/*w* biomass solution in 50 mM citrate-phosphate buffer (pH 5). Saccharification was performed at 50 °C with mixing at 180 rpm. Sampling was done in 3 h intervals until the maximum saccharification was achieved and then the slurry was centrifuged to separate the solids from the liquid. The liquid part was used for sugar quantification by HPLC (PerkinElmer, Waltham, MA, USA) and then used as the cultivation medium for the growth of the yeast. Bradford protein assay was used to measure the concentration of total protein in the hydrolysate.

### 3.3. Yeast Strain and Growth Condition

The oleaginous yeast *R. toruloides* NCYC 1576, procured from the National Collection of Yeast Culture (Norwich, UK) was used in the present work. The yeast culture was maintained on YPD (glucose, 20 g/L; peptone, 20 g/L; and yeast extract, 10 g/L) agar plates, which were sub-cultured at least twice per month to maintain the strain fresh. For the seed culture, yeast was initially cultivated in YPD broth at 28 °C for 24 h and the cells were harvested followed by washing with sterile distilled water to remove the media components. A cell density of 6.5–7.8 × 10^8^ cells/mL was obtained by adding the appropriate amount of sterile distilled water which was then used as the seed culture.

### 3.4. Optimization of the C/N Ratio (g/g) for the Lipid Fermentation by Oleaginous Yeast

For the optimization of the lipid accumulation, a fixed amount of glucose (40 g/L) and xylose (20 g/L) were mixed with yeast nitrogen base (YNB) without ammonium sulphate and amino acids (0.76 g/L) and yeast extract (0.1 g/L). The amount of glucose and xylose (2:1) were chosen on the basis of the amount present in the BSG hydrolysate. The different C/N ratios (20 to 200, 300, 400 and 500) were then adjusted by varying the concentration of (NH_4_)_2_SO_4_. In all the trials, batch cultivations were performed in Erlenmeyer flasks (250 mL) at 25 °C in a rotary shaker at 180 rpm. The pH of the medium was adjusted to 5.5 prior to inoculation by using 2 N HCl and NaOH solutions. All the trials were performed in triplicates.

### 3.5. Batch Cultivation Experiments

To study the effect of BSG hydrolysate on the growth and lipid accumulation of *R. toruloides*, the hydrolysate was diluted with distilled water to obtain a hydrolysate containing 40 g/L glucose with the amount of xylose being 21.5 g/L under these conditions. Thereafter, BSG hydrolysate was supplemented with a basal medium including YNB without ammonium sulphate and amino acids (0.76 g/L), and yeast extract (0.1 g/L). The C/N ratio was adjusted to 20 and 500 g/g by adding the appropriate amount of (NH_4_)_2_SO_4_ as nitrogen source. The amount of protein present in BSG (90 mg/L) was also taken into account for the calculation of C/N ratio. Finally, the pH was adjusted to 5.5 prior to sterilization.

### 3.6. Analytical Methods

To analyze the morphological variations during lipid accumulation in the living cells of *R. toruloides* grown in glucose based medium and BSG, 10 µL cultures were drawn at different time intervals and the cells were visualized in a compound light microscope (Olympus, Hamburg, Germany). The growth of yeast cells was monitored by measuring the cell optical density (O.D.) at 600 nm with a Genesys 10 S, UV/visible spectrophotometer (Thermo Fisher Scientific, Waltham, MA, USA). In order to estimate the cell dry weight, 50 mL culture was harvested by centrifugation (8000 rpm for 10 min) and the obtained pellets were kept on preweighed filter paper. The filter paper along with biomass was oven dried overnight at 60 °C and weighed using an analytical balance. The cell dry weight of *R. toruloides* was expressed as g/L. The concentration of the sugars was determined by an HPLC equipped with a refractive index detector and a Biorad Aminex HPX-87N column (BioRad, Hercules, CA, US). The column was maintained at 85 °C and 0.01 M Na_2_HPO_4_ was used as the mobile phase at a flow rate of 0.6 mL/min. The sugar consumption (%) was calculated with the following equation:

(1) $$\;C=\frac{St1-St2}{St1}\;\times 100\;$$

where *C* was the amount of sugar consumed, *St*1 was the amount of initial sugar added (g/L) and *St*2 was the residual sugar left at each sampling time.

To determine the lipid concentration, the lipids were extracted by a rapid ultrasonication-microwave treatment described by Patel et al (2018) with minor modifications. Briefly, 50 mL of culture broth were transferred to Teflon-capped Pyrex tubes (16 × 100 mm), centrifuged at 10,000 rpm for 10 min and the supernatant was discarded. The pellet was washed several times with distilled water to remove the media components. The cells were then dried in an oven at 55 °C and mixed with 10 mL of chloroform: methanol (2:1; *v*/*v*), then sonicated at 40 Hz for 2 min followed by microwave treatment for 2 min at 800 W. The slurry was stirred for 30 min and filtered through a sintered glass funnel, followed by the addition of 5 mL 0.034% MgCl_2_ and centrifugation at 3000 rpm for 5 min. The upper aqueous layer was aspirated, and the bottom chloroform layer was transferred to a new screw cap test tube. The concentration of the lipids was then determined gravimetrically after solvent evaporation and expressed as gram per liter (g/L). The extracted lipids were analyzed for their lipid components by thin layer chromatography (TLC) with a protocol described by Patel et al (2018) [58] and transesterified into fatty acid methyl esters by using acid catalysts as reported in the protocol of Laurens et al. (2012) [59]. The composition of fatty acids was analyzed by GC-FID (Agilent, Santa Clara, CA, USA) equipped with the capillary column (Select FAME; dimensions 50 m × 0.25 mm ID and 0.25 μm film thickness). The biodiesel properties of the corresponding FAMEs were analyzed with the help of derived empirical formulas as described before [58,60].

### 3.7. Statistical Analysis

The data values are means ± standard deviation of three independent recorded values. One- way analysis of variance (ANOVA) using Microsoft Office Excel 2016 (Microsoft, Redmond, WA, USA) with *p* < 0.05 was used for data acceptance.

## 4. Conclusions

The aim of this work was to establish a process for lipid production by oleaginous yeast using BSG hydrolysate. To improve the release of sugars during enzymatic saccharification, BSG was pretreated with two different methods, namely microwave assisted alkaline pretreatment and organosolv pretreatment. Organosolv pretreatment resulted in hydrolysate with higher sugar concentration and for this reason was used for the yeast cultivation. The growth and lipid production of *R. toruloides* were initially assessed using synthetic media consisting of glucose and xylose as carbon source, where the effect of carbon-to-nitrogen ratio was optimized. Finally, the optimized conditions were used for the culture of oleaginous yeast on BSG hydrolysates at C/N 500, resulting in accumulation of lipids at 10.41 ± 0.34 g/L (lipid content of 56.45 ± 0.76%) and 18.44 ± 0.96 g/L of cell dry weight after 168 h of culture. The fatty acids obtained from this yeast grown on BSG hydrolysate show a similar profile to vegetable oils, which can be used as feedstock for biodiesel production. Moreover, the FAME profile of biodiesel obtained after growth on BSG hydrolysate satisfy the criteria set up by ASTM 6751-3 and EN 14214 for the use as transportation fuel.

## Figures and Tables

**Table 1:** Comparative study of compositional analyses of BSG from various sources.

Cellulose (% *w*/*w)*	Xylan (% *w*/*w*)	Protein (% *w*/*w*)	Lignin (% *w*/*w*)	References
21.73 ± 1.36	13.63 ± 0.82	24.69 ± 1.04	19.40 ± 0.34	[23]
0.3 (1→3; 1→4)-β-Glucan	22.5 (Arabinoxylan)	26.7	n.d.	[24]
13–21	21–27	10–18	12–16	[25]
38.88 ± 0.87	18.23 ± 1.17	12.54 ± 0.65	13.7 ± 0.98	This study

n.d. not determined.

**Table 2:** Fatty acids of corresponding FAME (fatty acid methyl ester) profiles obtained after transesterification of lipids extracted from oleaginous yeast grown on various substrates.

Fatty Acids (%)		Glucose C/N 20		Glucose C/N 500		Xylose C/N 20		Xylose C/N 500		Glucose + Xylose C/N 20		Glucose + Xylose C/N 500		BSG C/N 20		BSG C/N 500	
Saturated Fatty Acid (SFA)	(C_14:0_)	ND	21.67	0.98	30.60	ND	24.49	0.89	30.46	0.91	26.87	0.86	31.4	ND	20.07	1.00	33.10
	(C_16:0_)	13.33		21.52		15.36		21.52		16.37		20.96		14.25		21.50	
	(C_18:0_)	6.69		8.10		7.56		6.32		7.76		8.34		3.96		7.04	
	(C_20:0_)	ND		ND		ND		ND		ND		0.47		ND		2.39	
	(C_24:0_)	1.65		1.18		1.57		1.73		1.83		0.77		1.86		1.17	
Mono Unsaturated Fatty Acid (MUFA)	(C_18:1n9t_)	46.25	46.25	51.14	51.14	47.91	47.91	42.37	42.37	43.45	43.45	52.37	52.37	54.84	54.84	50.85	50.85
Poly Unsaturated Fatty Acid (PUFA)	(C_18:2n6c_)	17.97	20.52	11.60	14.44	15.80	18.48	12.30	15.3	23.95	7.19	10.24	14.31	20.63	24.84	12.14	13.97
	(C_18:3n3_)	1.43		1.60		1.70		1.77		1.96		2.31		3.45		0.29	
	(C_22:2_)	1.12		1.24		0.98		1.23		1.28		1.76		0.76		1.54	
Total Fatty Acids (%)		88.44		96.18		90.88		88.13		97.51		98.08		99.75		97.92	

ND—not detected.

**Table 3:** Assessment of biodiesel properties by empirical formulas.

Biodiesel Properties	Units	Glucose C/N 20	Glucose C/N 500	Xylose C/N 20	Xylose C/N 500	Glucose + Xylose C/N 20	Glucose + Xylose C/N 500	BSG C/N 20	BSG C/N 500	Biodiesel Standards	
										ASTM D6751 Limits	EN 14214 Limits
Long chain saturation factor	-	7.978	8.562	8.456	8.772	5.517	6.736	3.405	8.060	-	-
Oxidative stability, 110 °C	h	8.7	11.5	9.3	11.0	7.1	12.0	7.5	12.1	3 min	6 min
Density	g/cm^3^	0.77	0.85	0.80	0.77	0.84	0.85	0.86	0.85	-	0.86–0.90
Cold filter plugging point	°C	8.6	10.4	10.1	11.1	0.9	4.7	0.6	8.8	-	-
Cloud Point	°C	2.0	6.3	3.1	6.3	3.6	6.0	2.5	6.3		
Pour Point	°C	−4.6	0.0	−3.5	0.0	−2.9	−0.3	−4.1	0.0		
Cetane number	-	59.2	57.5	58.7	61.8	54.3	57.2	52.1	58.2	47 min	51 min
Kinematic Viscosity	mm^2^/s	3.4	3.9	3.5	3.4	3.7	3.8	3.8	3.8	1.9–6.0	3.5–5
Saponification value	mg KOH/g-oil	177.1	197.0	182.4	178.4	193.3	197.0	197.0	195.8	0.50 min	0.50 min
Iodine value	mgI_2_/100g	79.8	73.3	77.8	67.1	89.8	74.6	97.3	70.8	-	120 max
High heating value	MJ/kg	35.0	38.5	36.0	34.8	37.8	38.5	38.7	38.2	-	-

- = Not reported; Min = minimum; Max = maximum.

## Abbreviations

AMP	Adenosine monophosphate
*ASTM*	American Society for Testing and Materials
BSG	Brewers’ spent grain
CCR	Carbon catabolite repression
CDW	Cell dry weight
CFPP	Cold filter plugging point
C/N	Carbon/nitrogen
CN	Cetane number
DAG	Diacylglycerols
FAMEs	Fatty acid methyl esters
FFA	Free fatty acids
h	Hour(s)
LCSF	Long chain saturation factor
LD	Lipid droplets
MAG	Monoacylglycerols
MUFAs	Mono-unsaturated fatty acids
NREL	National Renewable Energy Laboratory
OD	Optical density
PUFAs	Poly-unsaturated fatty acids
SFAs	Saturated fatty acids
TAGs	Triacylglycerols
TLC	Thin layer chromatography
YPD	Yeast extract peptone dextrose
YNB	Yeast nitrogen Base
